# Toward Understanding the Environmental Risks of Combined Microplastics/Nanomaterials Exposures: Unveiling ZnO Transformations after Adsorption onto Polystyrene Microplastics in Environmental Solutions

**DOI:** 10.1002/gch2.202300036

**Published:** 2023-07-05

**Authors:** Miguel A. Gomez‐Gonzalez, Tatiana Da Silva‐Ferreira, Nathaniel Clark, Robert Clough, Paul D. Quinn, Julia E. Parker

**Affiliations:** ^1^ Diamond Light Source Didcot Oxfordshire OX11 0DE UK; ^2^ School of Health Professions Peninsula Allied Health Centre University of Plymouth Derriford Road Plymouth PL6 8BH UK; ^3^ Analytical Research Facility School of Geography, Earth and Environmental Sciences University of Plymouth Plymouth PL4 8AA UK

**Keywords:** microplastics (MPs), X‐ray absorption near‐edge structure (XANES) spectroscopy, X‐ray fluorescence (XRF) nano‐imaging, Zn speciation changes, ZnO nanomaterials

## Abstract

Over recent decades, there has been a dramatic increase in the manufacture of engineered nanomaterials, which has inevitably led to their environmental release. Zinc oxide (ZnO) is among the more abundant nanomaterial manufactured due to its advantageous properties, used for piezoelectric, semiconducting, and antibacterial purposes. Plastic waste is ubiquitous and may break down or delaminate into smaller microplastics, leaving open the question of whether these small polymers may alter the fate of ZnO through adsorption within aquatic media (tap‐water and seawater). Here, scanning electron microscopy analysis confirms the effective Zn nano/microstructures adsorption onto polystyrene surfaces after only 24‐h incubation in the aquatic media. After pre‐aging the nanomaterials for 7‐days in different environmental media, nanoprobe X‐ray absorption near‐edge spectroscopy analysis reveals significant ZnO transformation toward Zn‐sulfide and Zn‐phosphate. The interaction between a commercial ZnO‐based sunscreen with polystyrene and a cleanser consumer containing microbeads with ZnO nanomaterials is also studied, revealing the adsorption of transformed Zn‐species in the microplastics surfaces, highlighting the environmental relevancy of this work. Understanding the structural and functional impacts of the microplastics/ZnO complexes, and how they evolve, will provide insights into their chemical nature, stability, transformations, and fate, which is key to predicting their bioreactivity in the environment.

## Graphical abstract

The ability of polystyrene microplastics to sorb aged ZnO nanomaterials in relevant freshwater and seawater scenarios is investigated for the first time, including potential ZnO leaching from commercial sunscreens. The adsorbed nanomaterials are examined by X‐ray nano‐spectromicroscopy, revealing significant ZnO transformation toward Zn‐sulfide and Zn‐phosphate. These results are crucial for determining their fate and behaviour in the environment after disposal.

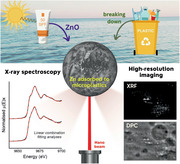

## 1 Introduction

Zinc oxide (ZnO) engineered nanomaterials (ENMs) are at the forefront of application‐driven nanotechnology due to their piezoelectric, semiconducting, and antibacterial properties, raising concerns about their potential impact as pollutants following their inevitable release into the environment. Sunscreens usually contain ZnO ENMs as the main active ingredient, to alleviate the risks associated with direct sun radiation onto the human skin, due to its great efficiency in absorbing UV radiation.^[^ ^1^ ^]^ Precisely, one of the primary pathways for environmental exposure to ZnO ENMs will be their subsequent disposal through drainage water (e.g., after swimming activities).^[^ ^2^ ^]^ The ZnO subproducts will then be processed through municipal sewage systems,^[^ ^3^ ^]^ ultimately ending up being incorporated into seawater.^[^ ^4^ ^]^

Likewise, plastic pollution has become a pressing environmental issue because the rate of disposable plastic manufacture has overcome our ability to deal with and recycle it efficiently. As an example, plastics are estimated to account for about half (≈54%) of anthropogenic waste materials released to the environment,^[^ ^5^ ^]^ due to a rise in its production from 1.7 million tons in 1950 to 368 million tons in 2019. In the absence of appropriate waste management infrastructure improvements, this is expected to increase by an order of magnitude by 2025.^[^ ^6^ ^]^ Once released to freshwater and seawater, UV‐exposure and mechanical abrasion can delaminate and degrade specific plastics, triggering their fragmentation into small plastic debris and microplastics (MPs).

Given the huge disposal of both MPs and ENMs into the environment, studying the interactions of MPs/ENMs is an area of great concern in environmental impact (and risk) assessment.^[^ ^7^, ^8^ ^]^ Concisely, the ENMs can experience both spatial (i.e., aggregation, adsorption) and spectral (i.e., chemical speciation) modifications that can lead to different effects, which will ultimately affect their eco‐toxicity and hazardousness. It has been reported that the heterogeneous MPs surface allows the adsorption of organic chemicals and metals,^[^ ^9^ ^]^ due to their high surface area and the variety of functional groups,^[^ ^10^, ^11^ ^]^ potentially acting as contaminant vectors and allowing the co‐exposure of more toxic substances. In addition, the nanometric size and the high surface reactivity of ZnO ENMs might not only increase their toxicity to the environment, but their reactivity toward the coexisting MPs. Thus, there is a concomitant need to predict the lifecycle and environmental implications of these combined nano‐ and micromaterials.

Even though there are several examples in the scientific literature of MPs transporting a range of pollutants,^[^ ^12^, ^13^, ^14^ ^]^ very few studies have focused on the transformations these nano‐ and micro‐pollutants may undergo when adsorbing to the MP surface. Even fewer have studied the interaction between microplastics and ZnO ENMs. Zheng et al.^[^ ^15^ ^]^ found that substantial apoptotic/distorted cells were observed in the different larvae‐tissues studied after either microplastics or ZnO ENMs individual addition and evidence of synergistic effects during co‐exposure. Estrela et al.^[^ ^16^ ^]^ also studied the effects of polystyrene (PS) nanoplastics (≈23 nm diameter, 760 µg L^−1^) after exposure to *Ctenopharyngodon idella*, both individually and in combination with ZnO ENMs. It is worth noting that no conclusive synergistic effects were found in this case, although all treatments containing "nanometric" PS instigated higher oxidative stress and DNA damage. The lack of combined effects suggests the adsorption dynamics between these nanopollutants are not fully resolved.

The aim of this work is to understand the influence of MPs as vectors for binding ZnO ENMs under different environmental conditions, which has had little investigation before. Studying how these ENMs transform when aged in freshwater and seawater after their disposal and revealing how they will interact with/adsorb to the microplastics at the nanoscale, are key for determining their fate and behaviour. Here, “real life” matrices (including the leaching of commercial sunscreens and exfoliating products) are also evaluated to identify the MPs/ENMs dynamics and the subsequent Zn‐speciation changes. The commercial sunscreen will act as a realistic source of ZnO nanomaterials to the environment, while a cleanser containing microbeads will mimic how microplastics may naturally be released (without added Zn‐components in their composition). With a beam size of ≈50 nm, the hard X‐ray nanoprobe at Diamond (I14) is a perfect fit for performing high‐resolution imaging via X‐ray fluorescence (XRF), also providing the spectral resolution needed to interrogate the Zn speciation using X‐ray absorption near edge structure (XANES) spectroscopy. In recent years, these techniques have emerged as promising methodologies for imaging environmental samples due to their high spatial resolution and sensitivity to a range of elements. They have been previously applied to study Zn accumulation and speciation within relevant biological^[^ ^17^ ^]^ and environmental samples.^[^ ^18^, ^19^ ^]^ XANES analyses have been used to understand how the ZnO ENMs alone transform and behave within wastewater treatment plant systems,^[^ ^20^, ^21^, ^22^ ^]^ but to date, no other author has attempted to investigate how the transformed Zn‐species and subproducts may absorb into a ubiquitous waste such as MPs. It is precisely the complexity of studying MPs’ interaction with co‐contaminants at the point of exposure, which makes it difficult to extrapolate conclusions widely. We propose to shed some light on this topic, by studying the polystyrene microplastic ability to sorb aged ZnO nanomaterials at the nanoscale, in relevant freshwater and seawater scenarios.

## 2 Experimental Section

### 2.1 ZnO Nanomaterials Characterisation and Commercial Products

Insoluble ZnO ENMs were purchased from US Nano (80–200 nm size, 99% pure). For characterisation purposes, 5 mg of ZnO were thoroughly mixed with 50 mL of Ethanol for 2 h. Subsequently, 20 µL of the above suspension were deposited on a holey carbon support copper grid (300 µm mesh, TAAB), leaving it to dry overnight inside a desiccator under vacuum. Transmission electron microscopy (TEM) and electron diffraction were performed on a JEOL STEM/TEM 2100Plus microscope, and the characterisation of the material is presented in Figure S1 (Supporting Information).

Pristine expandable polystyrene (PS) microplastics of ≈900 µm diameter were acquired from Goodfellow Cambridge, and pre‐characterised by SEM to ensure that no metallic Zn was present in the pristine product (data not shown). Polystyrene is one of the more commonly used and manufactured plastics, as well as one of the primary components of marine plastic debris,^[^ ^23^ ^]^ and hence it was selected as a model example.

A commercial sunscreen containing ZnO in its composition was also used. The brand of the commercial product was not indicated, but a table listing the sunscreen composition is presented in the Supporting Information (Table S1a, Supporting Information), in which non‐Nano ZnO is publicised as an active ingredient with an ≈18% content (as per manufacturers specifications).

Finally, an exfoliating cleanser listing microbeads as the main ingredient was purchased to act as a “real‐world” comparison of the pristine PS microparticles (Table S1b, Supporting Information).

### 2.2 Aging of ZnO Nanomaterials

Three aquatic solutions were selected as a case study mimicking the nanomaterials release into environmental media. Tap water from the I14 Chemistry Lab (Didcot, Oxfordshire, UK) was directly collected in February 2022, resembling one of the first mediums in which the ZnO nanomaterials from commercial products would be released after their primary use. In addition, two types of seawater were tested. The first was Instant Ocean tropic marin*. This artificial seawater in a dried salt form was mixed in the right proportion with ultrapure water (18 MΩ cm^−1^; Millipore Corp.), to create a synthetic medium simulating the marine environment without added dissolved organic matter.^[^ ^24^ ^]^ The second was seawater from the Mount Batten area (Plymouth), collected in January‐February 2022. The seawater was contained in holding tanks, and then biologically filtered and UV irradiated afterward, prior to use.

Separate suspensions of ZnO ENMs were made in each of the synthetic and environmental media above and two different sets were prepared at different ZnO ENMs mass concentrations: 100 and 250 mg L^−1^. Each suspension was left to age through continuous stirring (using a glass‐encased magnetic stirrer bar) for 7 days. This length was selected as a compromise to allow equilibrium conditions to be met. The aim was to allow the ZnO ENMs to stabilise, transform and undergo similar processes that they may face when released from commercial products (i.e., dissolution, aggregation).

### 2.3 Aging of Commercial Products

The commercial sunscreen containing ZnO was aged for the same period of time (7 days) in tap water and seawater, to evaluate how the active ingredient could potentially leach and subsequently transform in freshwater environments, after use and subsequent disposal. The ≈18% ZnO content listed in Table S1 (Supporting Information) was considered for adding a similar ZnO concentration (250 mg L^−1^) in the aging solution.

Furthermore, ≈1 g of exfoliating cleanser containing microbeads (Table S1b, Supporting Information) was also aged in 50 mL of seawater for 7 days, in combination with external ≈250 mg L^−1^ of ZnO ENMs, to mimic how these microbeads would naturally leach out after a week in seawater, assessing whether these microbeads could adsorb some ZnO onto the surface.

### 2.4 Determination of Total Element Concentrations after Aging

Following the 7‐day ZnO ENMs aging in the relevant media, samples of the tap water, artificial seawater, and seawater were filtered through 0.8 µm pore size membranes (MF‐Millipore) and then 10 mL were taken for total Cu, Fe, Ni, P, S, Ti, and Zn concentration using an ICP‐OES (iCAP 7400; Thermo Fisher). Prior to sample collection, individual 15 mL polypropylene centrifuge tubes were washed in 10% nitric acid overnight and then rinsed in ultrapure water to remove any residual elements of interest (e.g., total Zn). The tap water‐incubated water samples (including blanks) were analysed without any further dilution while the artificial seawater and seawater samples were diluted tenfold with ultrapure water in acid‐washed tubes (as above). The same procedure was also performed for the artificial seawater and seawater blank samples. In addition, two ultrapure water blanks were also analysed to determine, if any, their contribution to the total element measurements. The samples were not acidified prior to analysis due to the potential for trace element contamination. The concentration of each element in the samples was compared to standards of known concentrations (in 1% nitric for the tap water samples, or diluted seawater for both the artificial seawater and seawater samples). A 2% nitric acid wash solution was used in between samples to ensure minimal carryover. Yttrium was added to all standards and samples, to give a final concentration of 1 mg L^−1^, as an internal standard to account for instrumental drift and any variations in transport efficiency due to matrix differences. The limit of detection was calculated by the instrument software as three times the standard deviation of the blank signal.

### 2.5 Incubation of Aged Nanomaterials/Consumer Products with Microplastics

**Figure 1:**
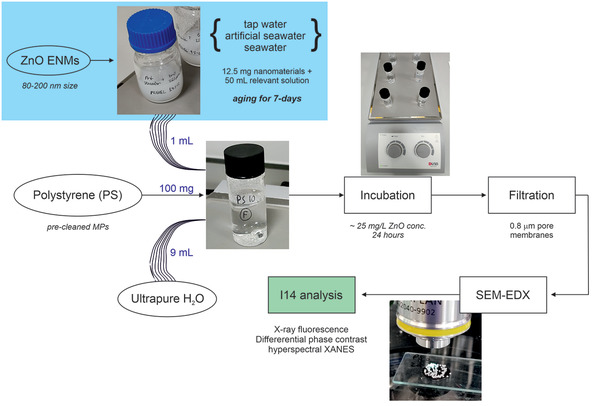
Summary of the experimental steps involving the ZnO engineered nanomaterials (ENMs) and the polystyrene (PS) microplastics, including the analysis carried out afterward. In brief, the ZnO ENMs were aged for 7‐days in relevant environmental media (blue‐squared region, Section 2.2), prior to mixing 1 mL of this solution with 100 mg of the polystyrene microplastics, together with another 9 mL of ultrapure water for incubation.

The PS MPs were pre‐cleaned with ethanol for 4 h and later left to dry in a fume hood. About 100 mg of PS were weighed in pre‐cleaned 15 mL glass flasks. Subsequently, 9 mL of ultrapure water were added together with 1 mL of the ZnO‐aged solutions listed in the sections above (either tap‐water, artificial seawater, or seawater) for a final ZnO concentration of ≈25 mg L^−1^ during the 24‐h incubation. The incubation procedure is summarised on the left side of **Figure** **1**.

A micrometric glass‐encased magnetic bar was used to leave the aged ZnO ENMs and the PS MPs under stirring for a fixed period of 24 h. After that, the incubation mixture was filtered through 0.8 µm pore‐size membranes (MF‐Millipore) using a ceramic funnel and a vacuum‐assisted glass filtration unit (Fischer Scientific UK). The PS MPs retained on the membrane surface were later rinsed with 1–2 mL of ultrapure water, and subsequently let dry in a glass Petri‐dish overnight (Figure 1).

### 2.6 Scanning Electron Microscopy Analysis

The filtered microplastics were deposited on carbon double‐sided tape and then glued into Al‐cylinder stubs (12.5×10 mm, Agar Scientific). A scanning electron microscope (JEOL JSM‐6610LV) was used to identify potential ZnO ENMs adsorption sites by scanning in backscattering mode (BEC) at 40 Pa. Energy dispersive spectroscopy (EDX) analyses were performed at 20 keV over the regions of interest for elemental quantification (*Zeiss*). Pristine MPs were initially inspected to ensure that no Zn‐background signal was present pre‐incubation.

### 2.7 Synchrotron X‐ray Nanoprobe Analysis

Nano‐focused X‐ray absorption near edge structure spectroscopy (XANES) and X‐ray fluorescence (XRF) analyses were carried out at the Hard X‐ray Nanoprobe at the I14 beamline (Diamond Light Source). The focused X‐ray beam size for this experiment was ≈60×60 nm. More details about the beamline optics and instrumentation are described elsewhere.^[^ ^25^ ^]^ The sample was raster scanned (continuous scanning) through the X‐ray focus, and the fluorescent X‐rays collected by a four‐element silicon drift detector (Rayspec) located in backscatter geometry, with a solid collection angle of 0.8 sr. A secondary Merlin Quad (Quantum Detectors, UK) photon counting detector was mounted in transmission geometry for differential phase contrast (DPC) imaging. Detailed information on how the transmitted signal was subsequently transformed is described by Quinn et al.,^[^ ^26^ ^]^ including masking of the beam, background intensity adjustment, and phase integration. A Lazic method was implemented, filtering the image to effectively suppress lower frequencies,^[^ ^27^ ^]^ for the final view of the DPC images.

The beam energy at the Zn edge was calibrated by measuring the first inflection point in the K absorption edge of a metallic Zn foil and adjusting this to 9659 eV. The XRF maps were acquired at 12 keV. PyMCA software was used to perform pixel‐by‐pixel background subtraction and to batch fit the fluorescence peaks, providing an image representing the distribution and concentration of each element present in the sample.^[^ ^28^ ^]^

Spectromicroscopy XANES analyses were performed by acquiring ≈150 independent XRF maps along the energies of the Zn K‐edge. An active drift compensation method was used to maintain alignment between successive scans. This process is automatic throughout the scanning at I14 and keeps the region of interest aligned to within a few pixels (irrespective of the scanning spatial size of the pixel) over the course of a XANES scan.^[^ ^29^ ^]^ Furthermore, the XANES maps were stacked, aligned, and normalised using the I_0_ intensity via in‐house python‐based scripts. Data visualisation, further alignment (if needed), and spectroscopic analysis were conducted using MANTiS.^[^ ^30^ ^]^ Principal components and cluster analysis were performed on the stacked XANES. Between 3 to 5 clusters grouping spectroscopically similar pixels were calculated by MANTiS, and their normalised XANES spectra were exported. Linear combination fitting (LCF) analyses of the normalised XANES region were performed by *Athena* from the Demeter software suite,^[^ ^31^ ^]^ comparing against the standard spectra of the expected Zn‐species previously described in wastewater systems, as follow: bulk ZnO, ZnO ENMs (80–200 nm, US‐Nano), ZnS, Zn_3_(PO_4_)_2_,^[^ ^22^ ^]^ ZnCl_2_ and a Copper Zinc Iron oxide (CuZnFe_2_O_4_) nanopowder (<100 nm, Merk). The LCF of the Zn spectra was carried out starting from the best fit with one component, and the number of components '*n*' was increased as long as the normalised sum of the squared residuals (NSSR = ∑(data_i_ − fit_i_)^2^/∑(data_i_)^2^) of best *n* + 1‐component fit was at least 10% lower than the NSSR of the best *n*‐component fit and if no component accounted for less than 5% of total Zn.

## 3 Results

### 3.1 Identification of Zn‐Particles Adsorbed to MPs Surfaces

**Figure 2:**
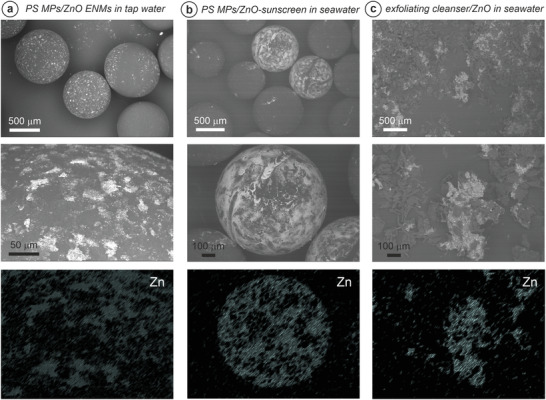
Scanning electron microscopy energy dispersive X‐ray spectroscopy (SEM‐EDX) for: a) the ZnO nanomaterials ENMs aged in tap‐water for 7 days, further incubated with polystyrene (PS) microplastics (MPs) for 24 h, b) the ZnO‐based sunscreen aged in seawater (7 days) and incubated with PS MPs (24 h), and c) the exfoliating cleanser incubated with ZnO ENMs in seawater for 7 days. In all cases, the top image is a low‐magnification view on the SEM, with the intermediate image zooming in the region subsequently analysed by EDX. The Zn‐signal from the EDX spectroscopy is displayed at the bottom after applying a different colour map and adjusting the histogram accordingly (no quantitative information is pursued from the EDX data).

Analysis using SEM allowed for a pre‐investigation of the isolated microplastics after incubation. Overall, the PS microplastics presented significant Zn adsorption over their surface, as confirmed by EDX (**Figure** **2**a). After aging the ZnO‐based sunscreen in seawater for 7 days and subsequently incubating an aliquot of that solution with the PS plastics for 24 h, some of the PS spheres presented a heterogeneous adsorption of Zn‐particles (Figure 2b). Aging the ZnO nanomaterials in seawater with the exfoliating cleanser containing microbeads for a week, favoured the presence of extended macro‐aggregates, which were deposited onto the filtration membrane. Irregularly shaped microplastics of different sizes were leached from the exfoliating cleanser after the incubation, with most of them presenting considerable Zn‐adsorption (Figure 2c).

### 3.2 Quantification of the Solutions after Aging and Incubation

The elemental total zinc (Zn), iron (Fe), phosphorous (P), and sulfur (S) concentrations of the <0.8 µm fractions, after filtrating the ZnO/MPs incubations, were quantified by ICP‐OES. The results presented in **Table** **1** confirmed that the “insoluble” ZnO nanoparticles were not extensively dissolved after the aging + incubation steps in the studied aquatic media, with a maximum of 3.5 µg L^−1^ of Zn found in the <0.8 µm fraction for the seawater. A maximum concentration of ≈43 µg L^−1^ total Zn was leached from the ZnO‐based sunscreen in seawater after 7 days, but this value should be considered negligible given the theoretical ≈250 mg L^−1^ ZnO allowed to age in the first place (taking into account that ≈18% of the sunscreen is ZnO according to its composition—Table S1a, Supporting Information). The lower amount of Zn leached from the same sunscreen in tap water (≈0.98 µg L^−1^), in comparison with the seawater (≈43 µg L^−1^), may be explained by the presence of more complex matrix on the seawater (e.g., containing different organic matter) which could potentially favour the leaching of ionic Zn. Nonetheless, and overall, these two values rule out the possibility that the non‐Nano ZnO from the sunscreen is effectively being dissolved as ionic Zn after 7 days of aging. Instead, it leaves open the question of what percentage of the remaining ZnO is to be found aggregated on the MPs’ surfaces or if, at the contrary, it had not been leached at all from the commercial sunscreen.

**Table 1:** Quantification of the dissolved fraction (< 0.8 µm) after incubation by Inductively Coupled Plasma Optical Spectroscopy (ICP‐OES)

	Elements quantified [µg mL^−1^] ^a)^			
Solution	Zn	Fe	P	S
Blank tap water	0.024	< LOD ^b)^	0.721	10.71
Blank artificial seawater	0.159	< LOD	0.011	483.0
Blank seawater	0.150	< LOD	0.105	704.9
ZnO 250 mg L^−1^ aged in tap water	0.093	0.013	1.507	11.19
ZnO 100 mg L^−1^ aged art. seawater	0.780	< LOD	< LOD	779.8
ZnO 100 mg L^−1^ aged in seawater	3.446	< LOD	< LOD	782.0
ZnO‐based sunscreen in tap water	0.985	0.014	0.649	12.266
ZnO‐based sunscreen in seawater	43.50	< LOD	< LOD	678.9

^a)^ Blank freshwater samples were analysed directly whilst saline and sunscreen in water samples were diluted 10‐fold with 2% HNO_3_ to reduce the sample matrix effects on the torch injector and plasma.

^b)^ Instrumental Limit of Detection (LOD) for all the elements measured was ranging from 0.002 to 0.017 µg mL^−1^, except for S in seawaters which was 3.0 µg mL^−1^. As the samples were diluted 10‐fold prior to analysis the minimum quantifiable concentration for each element was 10 times the LOD of the element.

### 3.3 Imaging the Distribution of Zn on the Polystyrene Surface

**Figure 3:**
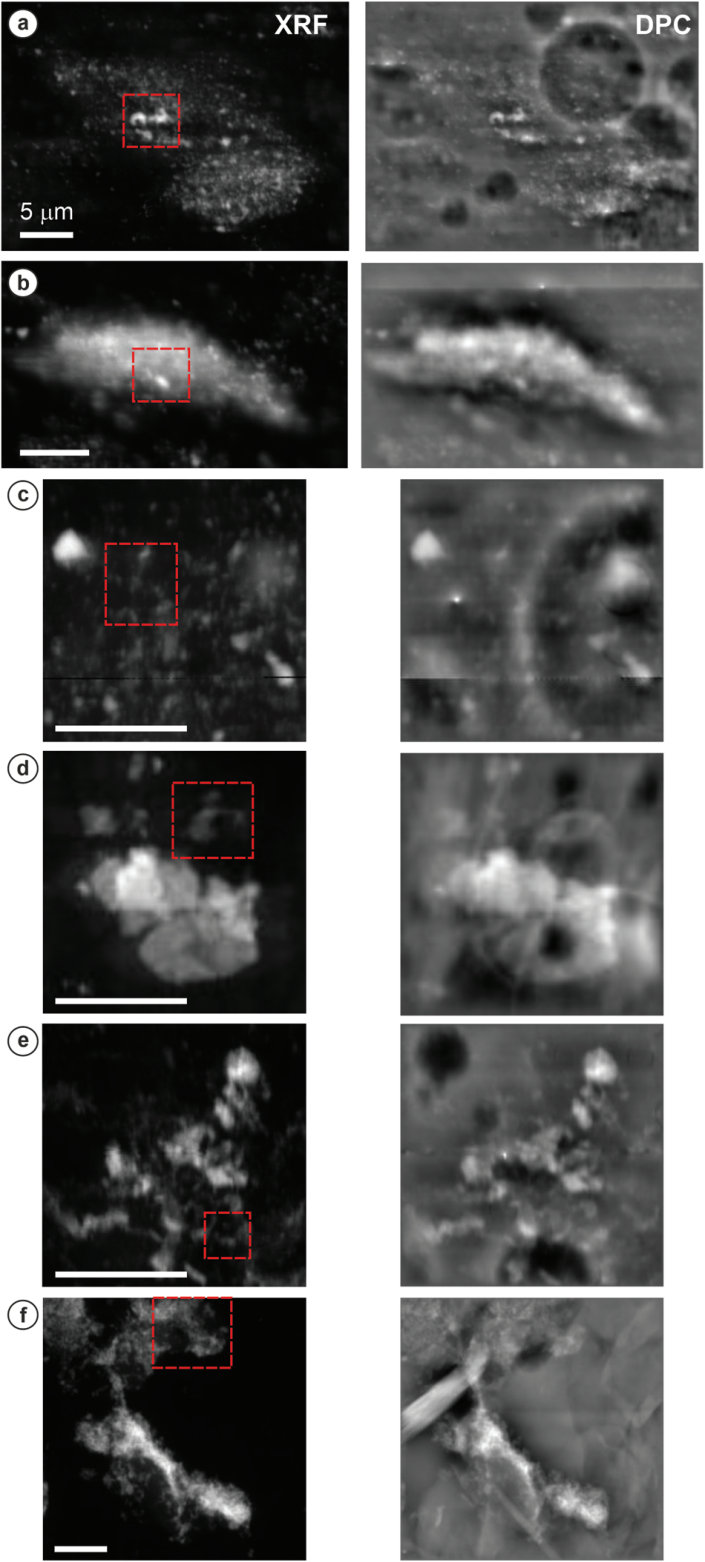
X‐ray fluorescence (XRF, left) maps (100 nm pixel size) for the Zn K‐edge and differential phase contrast (DPC, right) images—after applying a Lazic method to filter the lower frequencies from the signal (see Section 2.7 for details)—of Zn‐particles deposited on polystyrene microplastics using a) ZnO aged in tap water, b) ZnO aged in artificial seawater, c) ZnO aged in seawater, d) ZnO‐based sunscreen aged in tap water, and e) ZnO‐based sunscreen aged in seawater, as well as f) ZnO aged in seawater with the exfoliating cleanser. Red‐squared region indicates the areas where the XANES analysis was subsequently carried out (Section 3.4, Figure 4, Table 2). Scale bars = 5 µm in all cases.

Using the X‐ray Nanoprobe beamline, several high‐resolution maps (100 nm pixel size) were performed over a range of polystyrene/commercial microbead areas for all the conditions tested, as shown in **Figure** **3**. The X‐ray fluorescence (XRF) signal for the Zn K‐edge revealed the distribution of the adsorbed micro‐ and nanoparticles on the polystyrene surface after the incubation in different media (Figure 3a–c). In addition, the equivalent DPC images provided morphological information about the aggregates/clusters generated, as well as revealing the heterogeneities of the manufactured PS surface. Aging the ZnO ENMs in tap water maintained the adsorption of ZnO ENMs dispersed all over the microplastic surface, although some bigger aggregates (≈1–2 µm) were also found (Figure 3a). The ZnO ENMs aged in artificial seawater, on the contrary, showed macroscopic clusters deposited on the polystyrene surface after the 24 h incubation, which were composed of a combination of different micro‐particles together, as shown by the DPC image unveiled from the transmitted signal (Figure 3b). The ENMs kept in real‐world seawater presented a mixed behaviour though, with small particles deposited on the PS surface but also some bigger 2–3 µm aggregates (Figure 3c). When leaching the ZnO based sunscreen in tap water for 7 days, macro‐aggregates (>4 µm) of Zn were found adsorbed into the PS (Figure 3d), while leaching the sunscreen in seawater favoured the Zn distribution into smaller aggregates along the PS surface (Figure 3e). Looking at the irregularly shaped microplastics extracted from the exfoliating cleanser, the elemental Zn was spread on their surface at a significant rate, forming both micro‐ and macro‐aggregates (Figure 3f).

### 3.4 Zn Speciation after Aging in Freshwater and Seawater

**Figure 4:**
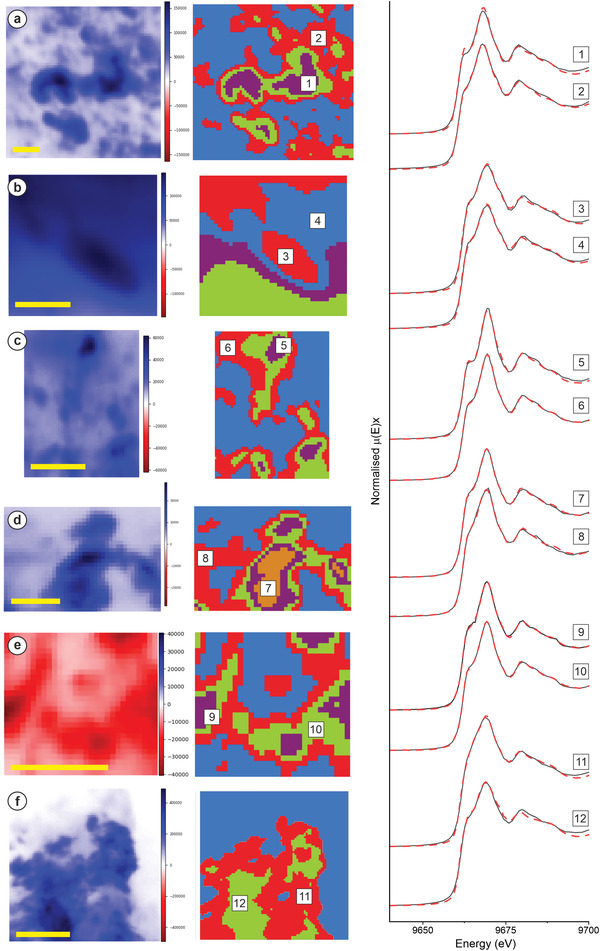
Principal component analysis [left] and cluster analysis [right] performed on the samples: a) ZnO/PS in tap water, b) ZnO/PS in artificial seawater, c) ZnO/PS in seawater, d) ZnO‐sunscreen/PS in tap water, e) ZnO‐sunscreen/PS in seawater, and f) exfoliating microbeads/ZnO in seawater. Averaged XANES spectra were extracted from relevant clusters (numbers in the white square) as follows: 1) violet cluster, sample a; 2) red cluster, sample a; 3) red cluster, sample b; 4) blue cluster, sample b; 5) violet cluster, sample c; 6) red cluster, sample c; 7) orange cluster, sample d; 8) red cluster, sample d; 9) violet cluster, sample e; 10) green cluster, sample e; 11) red cluster, sample f; 12) green cluster, sample f. These clusters were subsequently analysed by linear combination fitting (LCF) against the well‐known standards to interrogate the Zn speciation (see Table 2 for results). Scale bars = 1 µm in all cases.

One area from each high‐resolution XRF map was analysed by XANES mapping to interrogate the Zn‐speciation (red dashed‐square region, Figure 3). From the 3 to 5 cluster calculations provided by MANTiS spectromicroscopy software, only two distinct Zn spectra were selected per sample and subsequently analysed by LCF against the Zn‐standards available. The core of the big Zn‐aggregates adsorbed in the PS surface after incubation in tap‐water (violet color, cluster 1, **Figure** **4**a) maintained about 75% of ZnO composition, with ≈25% transformation toward Zn‐sulfide (**Table** **2**). The outside area surrounding the main particles (red, cluster 2, Figure 4a) presented a slightly lower percentage of ZnO (≈63%) but an increasing ≈20% contribution of Zn‐phosphate (Table 2). The two clusters of the Zn‐particles adsorbed into PS after incubation in artificial seawater were found to be very similar, in both cases the ZnO composition decreased to less than 50%, showing significant transformation to ZnS (35–38%) and Zn_3_(PO_4_)_2_ (clusters 3 and 4, sample b, Table 2). The incubation of ZnO/PS in seawater, however, caused less ZnO transformation, as demonstrated by the 77–88% contribution of ZnO remaining (clusters 5 and 6, Table 2).

**Table 2:** Linear combination fit results for the Zn K‐edge XANES spectra^a)^

Sample/cluster^b)^		ZnO	ZnS	Zn_3_(PO_4_)_2_	ZnCl_2_	CuZn‐Fe_2_O_4_	R factor ^c)^	red *χ* ^2^ ^d)^
		[%]						
*a*	1	75.2	23.7				0.00232	0.00063
	2	62.9	18.0	20.0			0.00046	0.00013
*b*	3	45.2	38.0	17.8			0.00131	0.00035
	4	48.4	35.6	16.9			0.00116	0.00031
*c*	5	88.4	11.8				0.00131	0.00038
	6	77.3	17.1		6.2		0.00021	0.00007
*d*	7	72.8	15.9			11.9	0.00044	0.00011
	8	67.6	15.7			18.0	0.00068	0.00024
*e*	9	72.6	13.7			14.3	0.00035	0.00010
	10	71.5	12.1			18.1	0.00030	0.00008
*f*	11	37.7	42.0	19.4			0.00299	0.00070
	12	46.1	32.2	21.9			0.00128	0.00030

^a)^ Zn K‐edge XANES spectra were analysed by LCF in the range: 9650 – 9700 eV. Fittings were not constrained to sum 100%.

^b)^ Sample descriptions as follows: a) ZnO/PS in tap water, b) ZnO/PS in artificial seawater, c) ZnO/PS in seawater, d) ZnO‐sunscreen/PS in tap water, e) ZnO‐sunscreen/PS in seawater, and f) exfoliating microbeads/ZnO in seawater. The cluster numbers indicate the regions analysed by LCF, as described in Figure 4.

^c)^ Normalised sum of the squared residuals of the fit [*R =* ∑(data‐fit)^2^ / ∑data^2^)]

^d)^ Goodness‐of‐fit was assessed by the *χ* ^2^ statistic [ = (*R* factor) / (no. of points – no. of variables)].

Looking at the commercial non‐Nano ZnO sunscreen, between 67% and 73% of the Zn‐structures found at the PS surface was effectively ZnO regardless of the incubation media (samples d and e, Table 2), while ≈12–16% of the Zn transformed toward ZnS, with a remaining 12–18% contribution of a mixture of Fe/Cu/Zn‐oxides. Finally, when incubating ZnO ENMs with exfoliating microbeads in seawater, they presented the lowest ZnO proportion of all the mixtures recorded (≈37–46%), ranging from 32% to 42% transformation toward Zn‐sulfide as well as 19% to 21% toward Zn‐phosphate (clusters 11 and 12, Figure 4f, Table 2).

## 4 Discussion

### 4.1 Effect of the Environmental Solutions on the ZnO Nanomaterials and in the Presence of Polystyrene

A combination of different sizes was found for the Zn‐based particles adsorbed onto polystyrene surfaces after aging/incubating the nanomaterials in the environmental media. Within tap water, a broad mixture was observed ranging from smaller individual nanoparticles to 1–2 µm‐sized aggregates. ZnO nanomaterials are prone to aggregation within stream water, especially at circumneutral pH,^[^ ^32^ ^]^ and this behaviour may have been enhanced during the aging process (7 days). The ENMs stability appears to be dictated not only by their physico‐chemical properties,^[^ ^33^ ^]^ but also by the chemistry of the dispersing solution. Incubating the ENMs in artificial seawater, for example, caused several microparticles to be closely combined when deposited in the polystyrene, forming macro‐clusters (Figure 3b). The higher ionic strength of this medium in comparison with the tap water would explain this tendency, due to the reduction of repulsion electrostatic forces between particles promoting aggregation^[^ ^34^ ^]^ and the increased presence of divalent cations.^[^ ^35^ ^]^ The ZnO aged in seawater was found adsorbed onto the PS surface in a wide range of sizes as well, from individual nanoparticles to larger 2–3 µm aggregates. Organic matter is set to play a role in the ZnO ENMs’ fate and stability, which will vary subject to its nature and aromaticity.^[^ ^36^ ^]^ Natural organic matter may stabilise nanomaterial suspensions,^[^ ^37^, ^38^ ^]^ by modifying the surface charge of the nanomaterials, but that would ultimately depend on the nanomaterials coating, and potential steric interactions/constraints.

Furthermore, ZnO ENMs dissolution could also play a significant role in the nanomaterials’ fate, especially when incubated in more acidic environmental media such as wastewater and sludge.^[^ ^39^ ^]^ Bian et al.^[^ ^40^ ^]^ indicated that there is a size‐dependent dissolution behaviour with the smallest ZnO nanoparticles showing a greater propensity for dissolution, hence the proven ZnO aggregation occurring during the aging and incubation procedures may have mitigated this conduct. In addition, ENMs/aggregates of different shapes (i.e., faceted, fibers, etc.) would present a less active surface, potentially hindering their dissolution. The fact that only ≈3.5 µg L^−1^ of Zn were quantified after filtrating the incubation solution through < 0.8 µm pore membranes (Table 1), supports the theory that minimum ZnO ENMs dissolution was occurring under the experimental conditions of this study, favouring the aggregation of the ZnO ENMs and their subsequent adsorption onto polystyrene surfaces. Chauque et al.^[^ ^41^ ^]^ found similar results in wastewater studies, reporting a decrease in the dissolved Zn concentrations when increasing both the ionic strength and the complexity of the aging/environmental media. It is also worth noting that the ZnO concentrations proposed in this study (100–250 mg L^−1^ during the aging, 10–25 mg L^−1^ when incubating with microplastics) are on the high‐end side of what would be environmentally relevant but within the nanoparticle concentrations range used in several bioavailability and toxicity studies (≈1–500 mg L^−1^).^[^ ^36^ ^]^ Lower concentrations of nanomaterials (in the µg L^−1^ range) may probably enhance the dissolution rates, limiting our ability to trace and detect the nanomaterials subproducts. Dissolution and adsorption kinetic studies may shed light on the mechanisms of these two processes and the competition between them, also offering relevant assessments of the associated environmental risks.

The XANES analysis allowed to further investigate the Zn speciation of the adsorbed particles/aggregates, which is required to infer their bioreactivity and potential toxicity. Aging the nanomaterials in tap water maintained ≈75% of ZnO, with the appearance of Zn‐sulfide in the core of the aggregates after their incubation with the polystyrene microparticles (Figure 4a). Nonetheless, this transformation transitioned considerably toward Zn‐phosphate speciation (≈20%) in the smaller and more isolated spots (red cluster, Figure 4a). The increment in Zn‐phosphate speciation compared to ZnS can be explained by the fact that transformation to Zn‐phosphate phases are thermodynamically favoured^[^ ^42^, ^43^ ^]^ while the formation of ZnS is kinetically preferred.^[^ ^44^ ^]^ The initial formation of ZnS species was also described in other scientific articles, either in stream^[^ ^3^ ^]^ or wastewater media,^[^ ^45^ ^]^ and in as little as a couple of hours.^[^ ^22^ ^]^

The Zn macro‐aggregates adsorbed into polystyrene after incubation in artificial seawater showed less ZnO importance (≈50%) than the same nanomaterials aged in tap water, presenting also significant transformation toward ZnS (35–38%) and Zn_3_(PO_4_)_2_ (17–18%) (clusters 3 and 4, sample b, Table 2). The dominant Zn‐speciation should be a function of the local microenvironments generated in the adsorbed structures onto the polystyrene, and higher ionic strengths may promote the ZnO transformation depending on the surface charges generated. Regarding why an increment of Zn‐sulfide species was found in comparison with the more thermodynamically stable Zn‐phosphate, it is also possible that indirect sulfiding can occur in the more sterically compromised macro‐clusters, limiting this transition, as reported during in situ incubations of ZnO nanorods.^[^ ^22^ ^]^ On the other hand, the ZnO/PS incubation in seawater maintained the highest percentage of ZnO (77‐88%), with only 11–17% of the zinc being transformed towards ZnS and a minor presence of ZnCl_2_ in the isolated areas. As explained above, the natural organic matter from the seawater may significantly impact the physico‐chemistry of the nanomaterials, which will ultimately affect their speciation. The formation of metal–organic complexes may partially block (sterically) the access to the metallic core of the nanomaterials, limiting its reactivity and bioavailability. These results highlight the need of applying characterisation methodologies such as the combination of nano‐ XRF/XANES, for gathering not only spatially but also spectrally resolved information, unveiling nanomaterials transformations, and speciation changes at relevant micro‐environments.

### 4.2 Transformed Zn‐Species Adsorbed to Microplastics—Environmental Risks

The described physico‐chemical and speciation changes of the nanomaterials may considerably impact their potential toxicity toward microorganisms. These two species present significantly lower solubility than ZnO, which varies depending on the crystallinity of the generated phases.^[^ ^44^ ^]^ Therefore, transformation toward Zn‐sulfide and Zn‐phosphate would limit the further release of ionic Zn into the medium. Poynton et al. studied the toxicity of these transformed species in sediment‐dwelling amphipods *Hyalella azteca*.^[^ ^46^ ^]^ In freshwater exposure, the phosphate‐aged particles were more toxic than other species, while the partially sulfidised ZnO nanoparticles presented two to five times lower toxicity (see Gomez‐Gonzalez et al.^[^ ^22^ ^]^ for a detailed discussion). Generally, increased sedimentation/precipitation of nanomaterials may also mitigate the ZnO ENMs toxicity by limiting their availability to the microorganisms. The adsorption of transformed Zn‐particles onto the polystyrene surfaces may have an opposite trend though, favouring the transportation of Zn nano/microaggregates and hence increasing their bioavailability to microorganisms and biota. The presence of polystyrene can induce different physico‐chemical changes to the ZnO ENMs according to Tong et al.,^[^ ^47^ ^]^ which was also related to the amount of UV‐vis radiation received. In “dark” conditions, limited ionic Zn was released from ZnO nanoparticles and these ions were not found to significantly adsorb into the polystyrene surface. Under sunlight irradiation, where more Zn^2+^ was detected in the medium, the polystyrene presence did not favour the transport of ionic Zn either, as the ZnO ENMs were mainly found as heteroaggregates, which co‐precipitated in natural waters. To the best of our knowledge, no scientific article has yet reported the presence of transformed Zn‐species adsorbed to microplastics’ surfaces, opening new investigation lines for accurately describing the bioreactivity and toxicity of these species in combination with microplastics.

### 4.3 Commercial Products—ZnO‐Based Sunscreen and Exfoliating Cleanser

Two commercial products were studied to provide a more realistic comparison with both the pristine polystyrene microspheres and the ZnO nanomaterials, as well as to extrapolate some conclusions to human exposure and health considerations. Macro‐aggregates (>4 µm) were found adsorbed on the pristine polystyrene surface following aging and incubation alongside the sunscreen in tap water, which was expected by the main ingredient description on the commercial product (Table S1a, Supporting Information), listed as non‐Nano ZnO, likely meaning that the original ZnO in the sunscreen was aggregated. Interestingly, aging the sunscreen into seawater favoured a more homogeneous leaching of ZnO ENMs, keeping the Zn‐particles more dispersed in comparison with the tap water, which settled/sedimented at a faster rate. As a consequence, incubating the sunscreen with the pristine microplastics in seawater promoted the distribution of Zn‐aggregates over the PS surface (Figure 3). As discussed in the previous sections, the presence of natural organic matter in seawater may be responsible for this conduct, by hindering the active ZnO ENMs active sites, affecting their aggregation behaviour during aging/incubation.

Regardless of the incubation media, the Zn‐particles adsorbed on PS after incubation presented an elevated contribution of ZnO (67–73%), together with a mixture of ZnS (12–16%) and CuZnFe_2_O_4_ (12–18%) (samples d and e, Figure 4, and Table 2). As shown for the ZnO ENMs incubated in tap water, a fair transformation toward Zn‐sulfide was described in all cases, confirming the “kinetic preference” of this phase under natural conditions. The complex matrix of the commercial product though, containing several surfactants and stabilisers in its composition (Table S1a, Supporting Information), might have had a substantial influence on the chemistry of the ZnO and subsequent transformation, which may also explain the copper zinc iron oxide presence. Unfortunately, no ICP‐OES quantification was carried out on the sunscreen, since the aim of this work was to investigate those consumer components readily desorbed/leached from incubation in natural waters, rather than performing a more comprehensive dissolution by microwave acidic digestion to interrogate its composition. ZnO structures with lengths ranging from 687 to 1512 nm are usually present in consumer sprays,^[^ ^48^ ^]^ and hence: i) the non‐Nano ZnO studied have environmental relevancy and ii) these results can be extrapolated to a range of products containing ZnO in their composition.

An exfoliating cleanser, listing microbeads in its product webpage description, was also assessed in this study to investigate the interaction of “real‐world” plastics leached from exfoliating products with pristine ZnO ENMs at seawaters according. Irregularly shaped microplastics were found in the filtrating membrane, after being leached from the exfoliating cleanser. These polymers adsorbed a range of Zn‐aggregates on their surface (Figure 3f). Looking at the XANES data, these complexes presented the lowest ZnO contribution (≈37–46%) from all the conditions studied, noticeably transforming toward Zn‐sulfide (32–42%) and Zn‐phosphate (19–22%). The Zn transformation enhancement may be explained by the number of surfactants present in the exfoliating cleanser (Table S1b, Supporting Information). It is worth mentioning that this product lists polyethylene (PE) as one of its components—and not polystyrene (PS)—which have different surface chemistry and reactivity.^[^ ^49^ ^]^ A few authors have studied the combined effects of PE and ZnO ENMs on different microorganisms and biota, finding mixed non‐synergistic^[^ ^50^ ^]^ and antagonistic^[^ ^51^, ^52^ ^]^ effects, hence not providing conclusive evidence of enhanced toxicity.

Further environmental processes, such as the aging of microplastics by UV–vis radiation (it has been reported that polymer surface textures were efficiently deteriorated after the addition of ZnO on polyvinylpyrrolidone under sunlight irradiation),^[^ ^53^ ^]^ and/or natural fragmentation/delamination of plastics in different shapes, would considerably affect their affinity toward ZnO or transformed Zn‐species. Hence, more specific (and tailored) investigations are needed, focusing on: 1) the different nature of microplastics available in consumer products, 2) the subsequent time‐dependent alterations these polymers may suffer when disposed of, and 3) how these alterations would affect the adsorption of ZnO ENMs.

## 5 Conclusions

The ability to image reacting ZnO nanomaterials in polystyrene microplastic surfaces has implications across research fields because it allows revealing the underlying pathways in which these ubiquitous wastes interact. This work demonstrates that a range of nano‐ and micro‐Zn aggregates are effectively found adsorbed to pristine polystyrene microplastics following incubation in freshwater and seawater solutions. The size and shape of the Zn‐aggregates strongly depend on the physico‐chemistry of the aging and incubating medium, with the natural organic matter from the seawater playing a significant role. Transforming Zn‐species were characterised by XANES spectroscopy at the nanoscale, overall showing mixtures of incipient Zn‐sulfide particles initially formed (kinetically favoured) and Zn‐phosphate in the more accessible sites (thermodynamically preferred).

The combined presence of polystyrene can induce different behaviour in the ZnO ENM subproducts, also acting as a transportation vector and potentially favouring internalisation inside living organisms.

In this study, two different consumer products: 1) a sunscreen containing “non‐Nano” ZnO, and 2) an exfoliating cleanser with microbeads in its composition, were evaluated under the same conditions, revealing that mixtures of Zn‐aggregates/micro‐polymers were naturally leached/released from the commercial products. Understanding how commercial ZnO nanomaterials transform in realistic hydrated media, and to what extent they adsorb onto the surfaces of ubiquitous microplastics waste, is key to carry out accurate environmental risk assessments. Defined and more comprehensive waste management regulations are needed for these MPs/ENMs since their presence in environmental scenarios will continue to (exponentially) grow. Given the persistent nature of these complexes, their threat to microorganisms and biota (and ultimately to the human‐chain food) cannot be ignored.

## Conflict of Interest

The authors declare no conflict of interest.

## Supporting information

Supporting Information

## Data Availability

The data that support the findings of this study are available from the corresponding author upon reasonable request.
