# Supplementary material for: Toward Understanding the Environmental Risks of Combined Microplastics/Nanomaterials Exposures: Unveiling ZnO Transformations after Adsorption onto Polystyrene Microplastics in Environmental Solutions
Source: Glob Chall. 2023 Jul 5;7(8):2300036. doi: 10.1002/gch2.202300036 (PMC10448137; doi:10.1002/gch2.202300036)
Supplement: Supplementary file 1 — Supporting Information [file GCH2-7-2300036-s001.pdf]

Global
Challenges
Open Access

Supporting Information

for *Global Challenges.*, DOI 10.1002/gch2.202300036

Toward Understanding the Environmental Risks of Combined Microplastics/Nanomaterials Exposures: Unveiling ZnO Transformations after Adsorption onto Polystyrene Microplastics in Environmental Solutions

Miguel A. Gomez-Gonzalez, Tatiana Da Silva-Ferreira, Nathaniel Clark, Robert Clough, Paul D. Quinn and Julia E. Parker*

Electronic Supplementary Information

Towards understanding the environmental risks of combined microplastics/nanomaterials exposures: Unveiling ZnO transformations after adsorption onto polystyrene microplastics in environmental solutions

Miguel A. Gomez-Gonzalez [a*], Tatiana Da Silva-Ferreira [a], Nathaniel Clark [b], Robert Clough [c], Paul D. Quinn [a], Julia E. Parker [a]

[a] *Diamond Light Source, Didcot, Oxfordshire, OX11 0DE, UK*

[b] *School of Health Professions, University of Plymouth, Peninsula Allied Health Centre, Derriford Road, PL6 8BH, UK*

[c] *Analytical Research Facility, School of Geography, Earth and Environmental Sciences, University of Plymouth, Plymouth, UK*

*corresponding author: miguel.gomez-gonzalez@diamond.ac.uk

(1 Figure, 1 Table)

Table of Contents

S1. Characterisation of ZnO engineered nanomaterials S2

S2. Commercial products' composition S3

S1. Characterisation of ZnO engineered nanomaterials

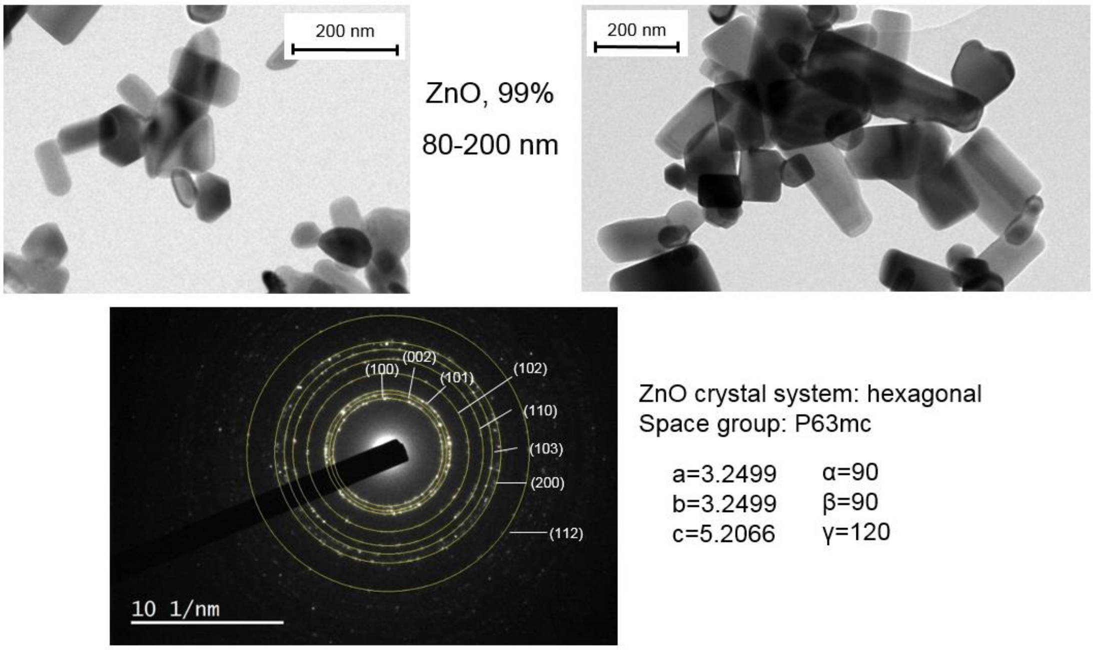

Figure S1. Commercial ZnO engineered nanomaterials (ENMs) characterised by transmission electron microscopy TEM and electron diffraction analyses (*US nano*, 80-200 nm size, 99% pure).

S2. Commercial products' composition

Table S1. Composition of the commercial products studied in this manuscript: a) sunscreen containing non-Nano ZnO and b) exfoliating cleanser containing microbeads.

**a.- non-Nano ZnO sunscreen**	
Active ingredients	
**ZnO**	**16 %**
Inactive ingredients	
Arachidyl alcohol, arachidyl glucoside, behenyl alcohol, bisabol, butyloctyl salicytate, butyrospemum parkii, caprylhydroxamic acid, caprylyl glicol, cellulose gum, cetearyl alcohol, cetyl alcohol, citric acid, coco-glucoside, glycerin, methyl dihydroabietate, microcristal-line cellulose, polyhydroxystearic acid, sodium gluconate, sodium hialuronate, sodium stearoyl, glutamate, tocopherol, water	
**b.- Exfoliating cleanser containing microbeads**	
Ingredients	
Gentle micro-beads for effective cleansing, Propylene Glycol, Sodium Laureth Sulfate, Polyethylene, Cocamidopropyl Betaine, Disodium Lauroamphodiacetate, PEG-120 Methyl Glucose Dioleate, Lauryl Methyl Gluceth-10 Hydroxypropyldimonium Chloride, Hamamelis Virginiana Distillate, Glycol Distearate, Farnesol, Glycerin, Laureth-4, Alcohol, Sodium Carbomer, Sodium Chloride, Tetrasodium EDTA, Lactic Acid, Sodium Benzoate, Methylparaben, Parfum	
